# Perspectives in Searching Antimicrobial Peptides (AMPs) Produced by the Microbiota

**DOI:** 10.1007/s00248-023-02313-8

**Published:** 2023-12-01

**Authors:** Luigui Gallardo-Becerra, Melany Cervantes-Echeverría, Fernanda Cornejo-Granados, Luis E. Vazquez-Morado, Adrian Ochoa-Leyva

**Affiliations:** Departamento de Microbiologia Molecular, Instituto de Biotecnologia, Universidad Nacional Autonoma de Mexico (UNAM), Avenida Universidad 2001, C.P. 62210 Cuernavaca, Morelos Mexico

**Keywords:** Antimicrobial peptides (AMPs), Microbiota, Microbiome

## Abstract

Changes in the structure and function of the microbiota are associated with various human diseases. These microbial changes can be mediated by antimicrobial peptides (AMPs), small peptides produced by the host and their microbiota, which play a crucial role in host-bacteria co-evolution. Thus, by studying AMPs produced by the microbiota (microbial AMPs), we can better understand the interactions between host and bacteria in microbiome homeostasis. Additionally, microbial AMPs are a new source of compounds against pathogenic and multi-resistant bacteria. Further, the growing accessibility to metagenomic and metatranscriptomic datasets presents an opportunity to discover new microbial AMPs. This review examines the structural properties of microbiota-derived AMPs, their molecular action mechanisms, genomic organization, and strategies for their identification in any microbiome data as well as experimental testing. Overall, we provided a comprehensive overview of this important topic from the microbial perspective.

**Supplementary Information:**

The online version contains supplementary material available at 10.1007/s00248-023-02313-8.

## Introduction

The role of the microbiota is crucial for maintaining good health. It helps to develop the host’s physiology, protects against harmful pathogens, and regulates metabolic processes [1]. Moreover, the microbiota also produces metabolites that can affect the host’s homeostasis [2]. For instance, short-chain fatty acids (SCFA) produced by some bacterial species play a vital role in cross-communication, mucus barrier [3], gut motility [4], blood pressure regulation [5], bile acids deconjugation [4, 5], amino acid production [6], and vitamin synthesis [7]. Thus, understanding how the microbiome is self-regulated and modulated opens new possibilities for microbiome-based therapies via microbiome engineering [8].

The proteins excreted and secreted by the microbiota, also known as the secrebiome [9], play a crucial role in the communication between the microbiota and their host. These proteins include enzymes, toxins, and antimicrobial peptides (AMPs) [10, 11]. AMPs are an ancestral and effective primary defense mechanism against pathogens such as bacteria, archaea, fungi, and viruses [11]. They do not have enzymatic activities and can act in a monomer or polymer conformation [10, 11]. Also, these peptides autoregulate bacteria, conducting communication with each other through quorum sensing [12], as well as with eukaryotic host cells [13], and regulate virulence systems [14]. In this regard, the microbiota and their host can produce AMPs for microbiota-microbiota or microbiota-host interactions. This review will focus specifically on microbiota-derived AMPs, their general properties, molecular action mechanisms, and strategies for their identification in any microbiome data set, and experimental validation.

## General Properties of AMPs

**Fig. 1:**
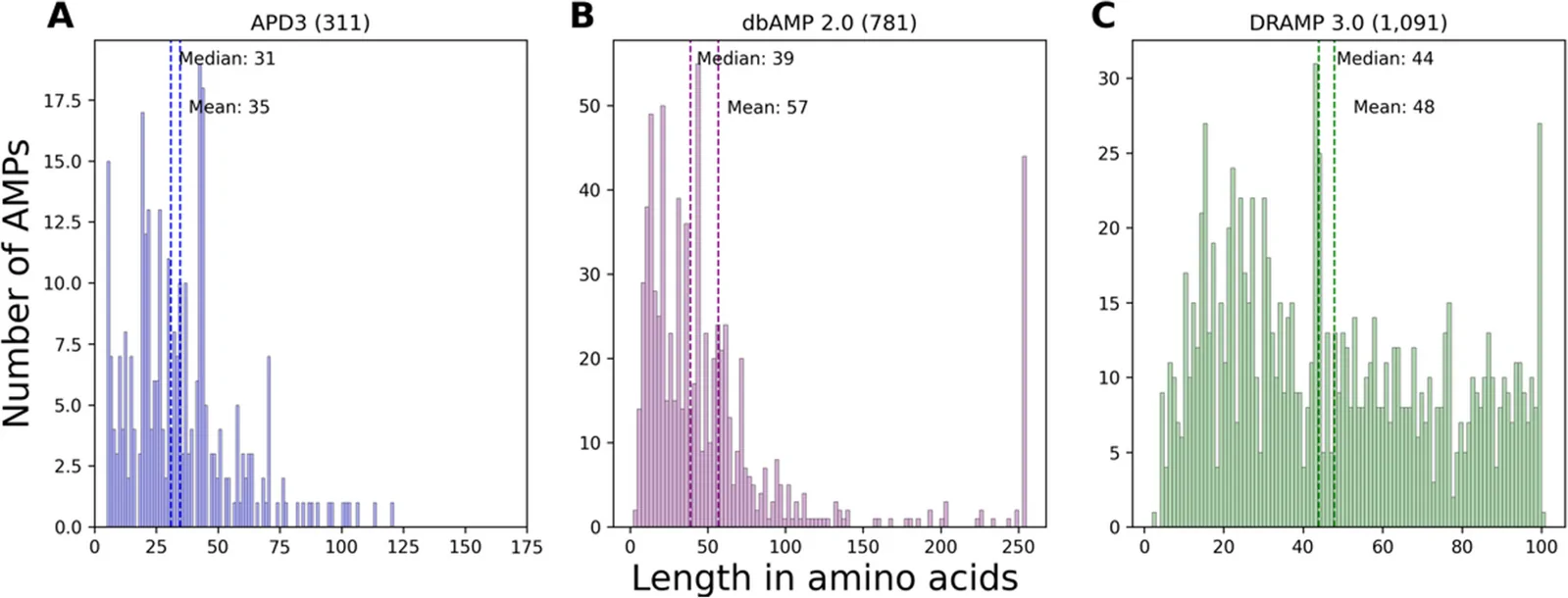
Length distribution of microbial AMPs deposited in databases. **A** APD3, an experimentally validated database with 311 AMPs produced by prokaryotes, had a median of 31 aa, and a mean of 35 aa; **B** dbAMP 2.0, a collection of experimentally validated and hypothetical AMPs, had 781 peptides produced by microorganisms with a median of 39 aa, and a mean of 57 aa; **C** DRAMP 3.0, also a collection of validated and putative AMPs, contained 1091 peptides from microbial origin, with a median of 44 aa, and a mean of 48 aa

It is well-known that AMPs have low molecular weight and minimal secondary structure compared to regular proteins [15]. These molecules are typically cationic and amphipathic, containing both hydrophobic and hydrophilic regions. They can adopt various conformations, such as α-helical, ß-sheet, or extended (without a specific structural motif). Most AMPs reported in publicly available databases are short peptides, with a typical length ranging from 5 to 50 amino acids [16]. However, some peptides are longer with over 200 aa [17, 18]. To better understand these characteristics, we analyzed the peptide length distributions in three AMP databases: APD3, dbAMP, and DRAMP, which contain both predicted and experimentally tested AMPs. Our analysis found that all AMPs in these databases had a mean of 33–40 amino acids (Supplementary Fig. 1). On the other hand, when we focused only on microbiota-derived AMPs, we observed these peptides had a mean length from 35 to 57 amino acids (Fig. 1).

**Fig. 2:**
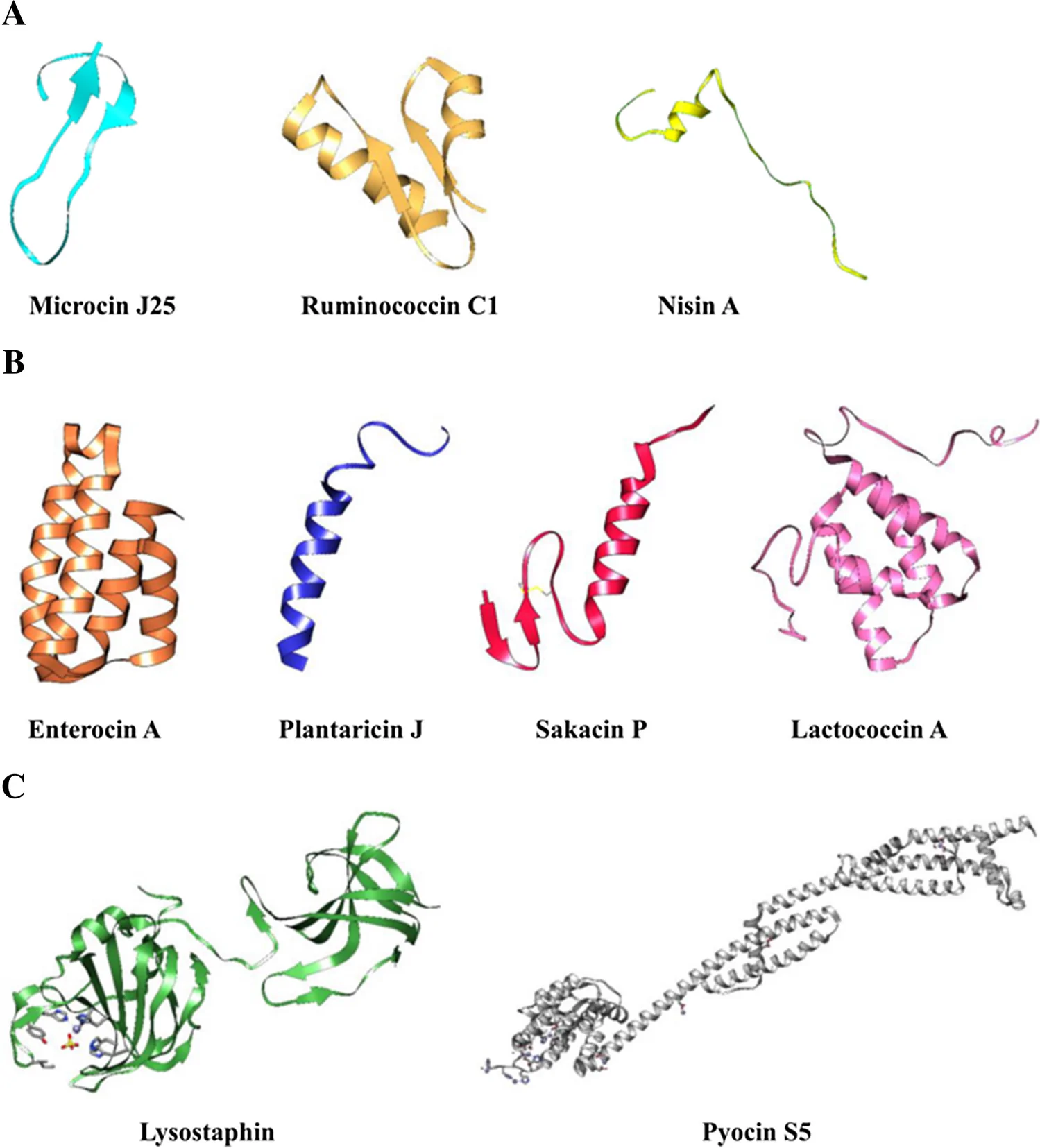
Three-dimensional structure of the three classes of AMPs. **A** Class I AMPs, which are bacteriocins-like peptides include Microcin J25 (1Q71), Ruminococcin C1 (6T33), and Nisin A (1WCO). **B** Class II AMPs include pediocin-like peptides such as Enterocin A (2BL7), Plantaricin J (2KHG), Sakacin P (1OG7), and Lactococcin A (5LFI). **C** Class III AMPs have bacteriolysin activity, for example, Lysostaphin (4LXC) and Pyocin S5 (6THK). Each class has unique characteristics and plays a crucial role in fighting against harmful microbes. The Protein Data Bank (PDB) identifier of 3D structures is mentioned in parenthesis

Based on their structure, microbial AMPs can be categorized into three groups (Fig. 2) [19]. Class I, with peptides of less than 10 kDa with a similar structure to Microcins (Fig. 2A). These peptides are composed of two alpha helices, such as Glycosin F, two beta sheets as in Microcin J25 (Fig. 2A), or a combination of both, which fold the peptide into a horseshoe shape, and at the N-terminal or C-terminal ends, peptide segments can be found without a given conformation, as seen in Ruminococcin C (Fig. 2A)[19–21]. Class II includes peptides larger than 10 kDa and structures like Pediocins (Fig. 2B). These peptides are made up solely of alpha helices that occupy most of the sequence. Lacticin Q and Plantaricin J are among its members (Fig. 2B) [22–25]. Finally, Class III comprises peptides about 30 kDa in size, such as Bacteriolysins (Fig. 2C). These peptides exhibit a structural complexity comparable to native proteins and can be conjugated with other ions (Fig. 2C) [19, 26–28].

## Microbial AMPs’ Molecular Action Mechanisms

**Fig. 3:**
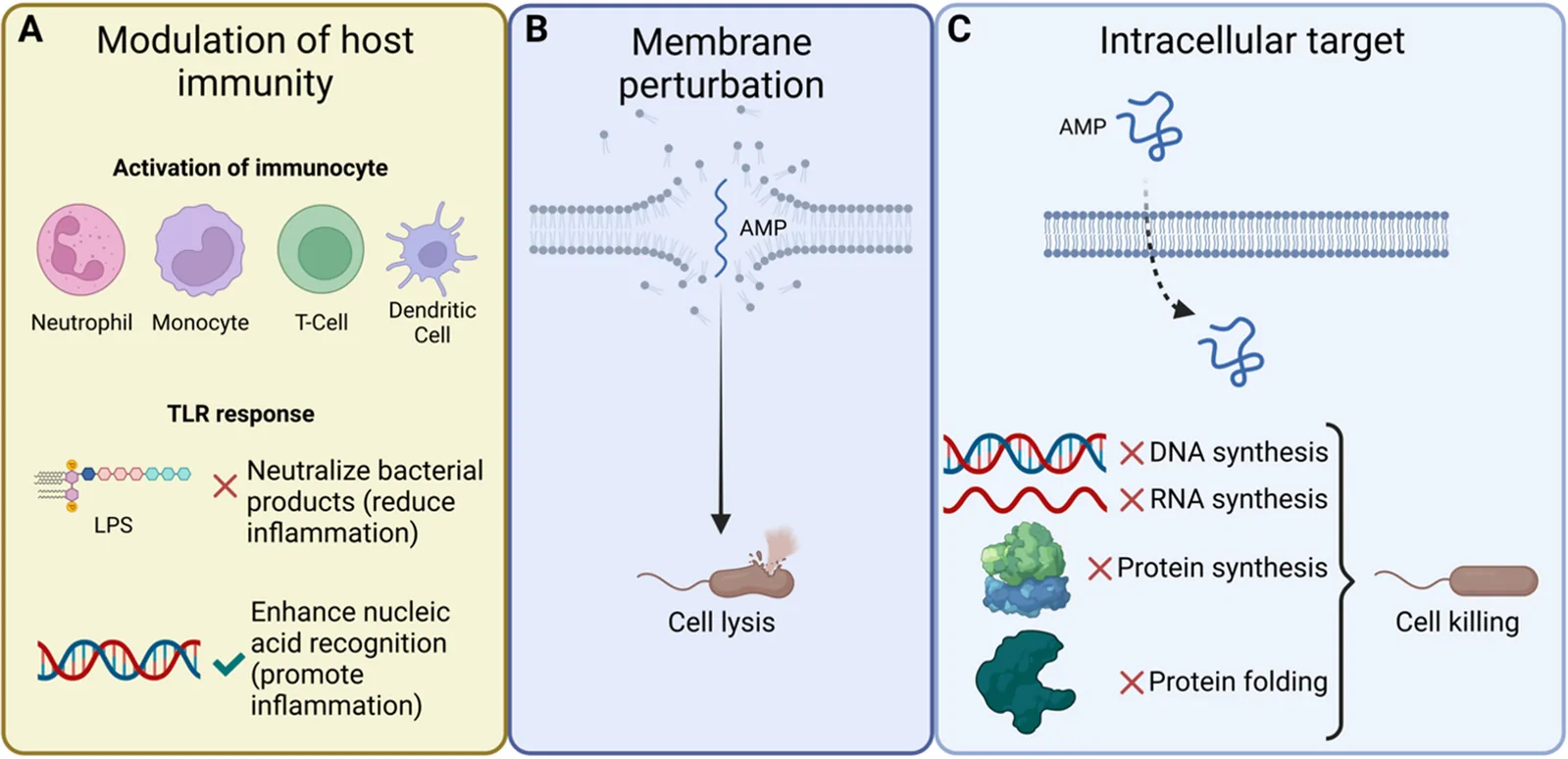
Microbial AMPs action mechanisms. **A** AMPs significantly impact host immunity by activating the immune cell response or toll-like receptors (TLR). This activity is essential because it can neutralize bacterial products such as lipopolysaccharides (LPS), thus reducing inflammation or enhancing microbial nucleic acid recognition, thereby increasing inflammation. Furthermore, AMPs can directly cause the death of the target microorganisms, either **B** by perturbing its membrane, leading to cell lysis, or **C** by inhibiting vital intracellular functions. These makes AMPs a potent weapon in the fight against harmful bacteria. The image was created using BioRender.com.

Regarding their biological mechanisms, microbial AMPs can be classified into three groups (Fig. 3). The first includes AMPs that play a regulatory role in the host's immune system. They achieve this by activating and recruiting immunocytes or altering the Toll-like receptor (TLR) recognition of microorganisms (Fig. 3A) [16]. The second group includes AMPs that interact with the membrane or cell wall of the target microorganism, causing lysis (Fig. 3B). The selectivity of these AMPs depends on specific differences in the composition of the membrane or cell wall [11, 16] and can be further classified into barrel-slave [29], carpet-like [30], and toroidal pores [31]. Finally, the third group consists of AMPs that inhibit essential intracellular functions, such as DNA replication (Fig. 3C) [16, 32]. It is worth noting that all three mechanisms are shared between AMPs produced by bacteria, fungi, and protozoans [33, 34].

**Fig. 4:**
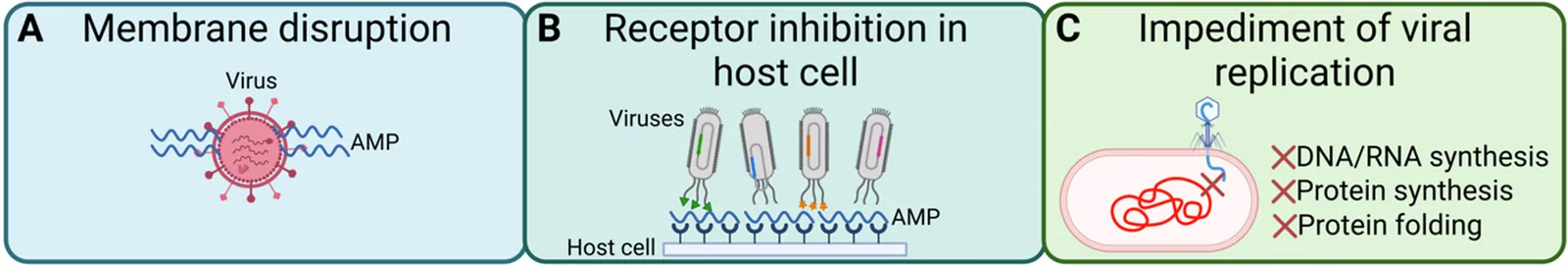
Action mechanisms of antiviral peptides. Antiviral peptides work by inhibiting the viability of the virus, preventing the infection of target cells, or impeding the virus’s ability to replicate. There are several mechanisms by which these peptides work including **A** the direct disruption of the viral membrane, making the virus unable to infect, **B** blocking the viral receptor binding to the host cells, thus preventing the virus from entering the target cell **B**, and **C** blocking intracellular functions necessary for viral replication **C**. Image created using BioRender.com

Microbial AMPs can have additional benefits as antiviral peptides, inhibiting the virus viability [35]. These peptides are effective against DNA and RNA viruses (Fig. 4) and work by disrupting the viral membrane to prevent host cell infection (Fig. 4A) [35]. Other mechanisms include inhibiting the host viral receptor (Fig. 4B) [36], as well as targeting specific intracellular molecules required for viral replication (Fig. 4C) [37]. These findings represent potential alternatives for developing new antiviral treatments based on AMPs.

## Classic Production and Genomic Organization of Microbial AMPs

**Fig. 5:**
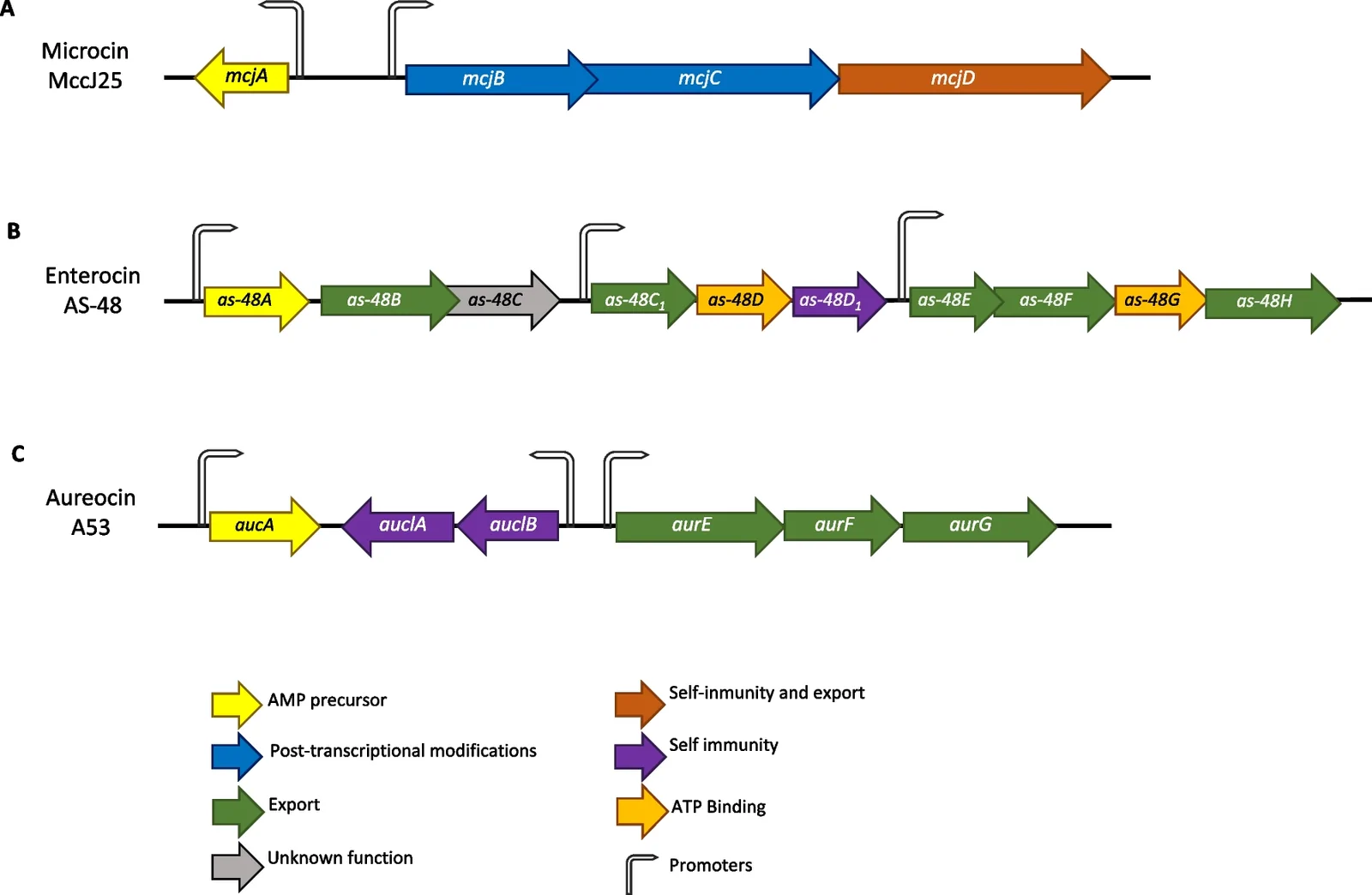
Examples of the genomic organization of microbial AMPs gene clusters. Arrows indicate the genes colored based on their known or putative functions. A flag indicates promoters and the direction refers to gene transcription. **A** shows the genes required for microcin MccJ25; **B** shows the genes required for Enterocin AS-48, and **C** exhibits genes required for Aureocin A53

AMPs are produced through the activation of genes responsible for controlling AMP synthesis. These genes are typically stimulated by infectious or inflammatory processes [38] and are commonly organized in a single or several operons. These operons include one or more structural genes encoding a functional peptide or their inactive precursor and genes for AMP regulation, maturation, export, and self-immunity, typically adjacent in the cluster arrangement [38]. The genetic organization varies among bacterial AMPs. For instance, the cluster of the microcin MccJ25 gene of *Escherichia coli* is partially conserved, including at least one precursor, self-immunity against the AMP, and export genes (Fig. 5A).

On the other hand, enterocin AS-48 produced by *Enterococcus faecalis* may require at least five to seven genes for its production and autoimmunity (Fig. 5B) as well as an accessory operon encoding the ABC transporter protein complex [39]. Complex AMPs, such as lantibiotics produced by Gram-positive bacteria, can undergo post-transcriptional modifications before being secreted. Usually, clusters carry the genes to produce the enzymes responsible for post-transcriptional modification, while other AMPs are translated and exported without modifications [40]. However, there are clusters for some AMPs, such as aureocin A53 of *Staphylococcus aureus*, that do not include genes for post-translational processing (Fig. 5C) [41]. Furthermore, large AMP operons often carry one or more genes of unknown or uncharacterized function [39]. The activation of secretion system genes, typically ABC transporters, is responsible for AMP export from the cytosol to the extracellular environment, completing the production of AMPs [42].

## Microbial AMPs in the Microbiome Regulation

All higher organisms have a close relationship with the microbiota inhabiting them. AMPs, which both the host and the microbiota produce, are essential for crosstalk communication and maintaining the homeostasis of the microbiome. Additionally, AMPs are part of the first line of the host defense by inhibiting the proliferation of potentially harmful pathogens [43, 44]. On the other hand, microorganisms use them to take advantage of an environmental niche by manipulating the host or competing with other microorganisms within the microbiota [16, 44]. AMPs can also regulate species-specific associations and can influence bacterial colonization [45].

**Fig. 6:**
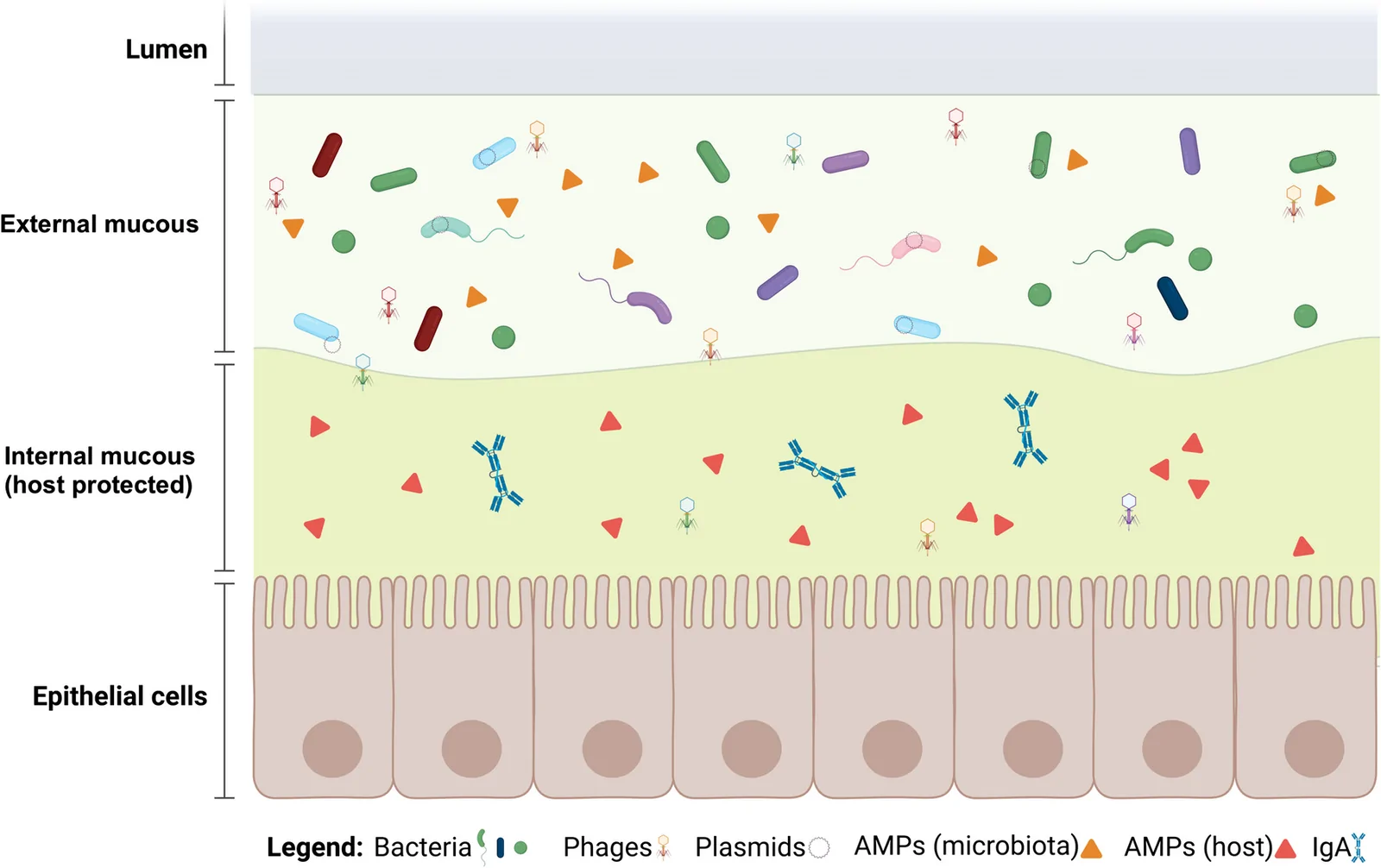
Illustration of the critical role played by AMPs in the gut microbiome dynamics. AMPs play an essential role in the ecological dynamics of the gut microbiome. The host’s AMPs secreted in the gut ensure that the internal mucosa remains uncolonized by potentially harmful bacteria. At the same time, the AMPs produced by the microbiota compete with each other and regulate the host’s response to them. Moreover, AMPs can be carried by mobile genetic elements such as bacteriophages and plasmids, which can confer an advantage to their hosts in the microbiome dynamics. The image was created using BioRender.com

The human gut is a fascinating and well-studied example of how microbes interact with their host, and one area that has received much attention is the interaction with microbial AMPs (Fig. 6). Although there is scarce information about the specific bacteria that produce AMPs, we do have some clues. Some of the most common producers of AMPs in the human gut microbiome are *Bacillus* and *Lactobacillus*, transient bacteria colonizing the epithelium [46]. These microbes are known for producing bacteriocins and lipopeptide antibiotics that suppress the growth of potential pathogens by affecting membrane permeabilization [47–49]. Also, administering probiotics that produce AMPs, such as members of *Lactobacillus* and *Enterococcus*, has improved antimicrobial activity in the intestinal lumen [50]. *Butyrivibrio* is another example of a microbe that produces AMPs and is found in high abundance in the intestine of mice after exercise-induced stress response [51]*.* Other types of peptides with antimicrobial activities include ribosomally synthesized and post-translationally modified peptides (RiPPs), such as lanthipeptides produced by Firmicutes and Actinobacteria, and sactipeptides, primarily characterized in *Bacillus* species, thiopeptides, reported in *Lactobacillus gasseri*, *Cutibacterium acnes*, *Enterococcus faecalis*, *Streptococcus downei*, and, *S. sobrinus* [2]. It is also worth noting that host intestinal epithelial cells produce AMPs, particularly bacteriocins, lanthipeptides, and sactipeptides, that control the overgrowth of unwanted bacteria in the inner mucus layer [52]. Conversely, the microbiota produces AMPs to compete for gut establishment [16].

The microbiota’s mobile genetic elements, including bacteriophages and plasmids are carriers of AMPs enhancing the carrier microorganisms’ fitness [53–55]. A significant fraction of AMPs, such as microcins, are usually transported by conjugative plasmids, allowing for their exchange between bacteria. A well-described example is the microcins MccB17 produced by certain strains of *Escherichia coli*, which carry the 70 kb conjugative plasmid pRYC17 with gene clusters to produce precursors, post-transcriptional enzymes, secretion, and autoimmunity genes [56]. Some lytic phages have cell wall hydrolytic AMPs, such as lysins and lysozymes, which form a hole in peptidoglycan structure and release replicated viral particles. Purified phage endolysins have been applied against Gram-positive pathogens such as Streptococcus pyogenes as potential antimicrobial agents [57, 58].

The microbiota is a valuable source of compounds for the industry and a promising source of novel biomolecules like AMPs [14]. However, most microorganisms cannot be cultured, making DNA and RNA sequencing viable alternatives for discovering AMPs in microbial communities. This method also allows us to study AMPs produced by the microbiota without the need to culture the original bacteria. Unfortunately, few bioinformatics protocols are available to identify AMPs in DNA or RNA sequencing datasets. In the next section of this review, we explore the current state of bioinformatic tools for discovering AMPs from the microbiota. Also, we summarize some of the most successful experimental strategies for the functional analysis of AMPs.

## Genomic Sciences Applied to Microbiomes to Discover AMPs

**Fig. 7:**
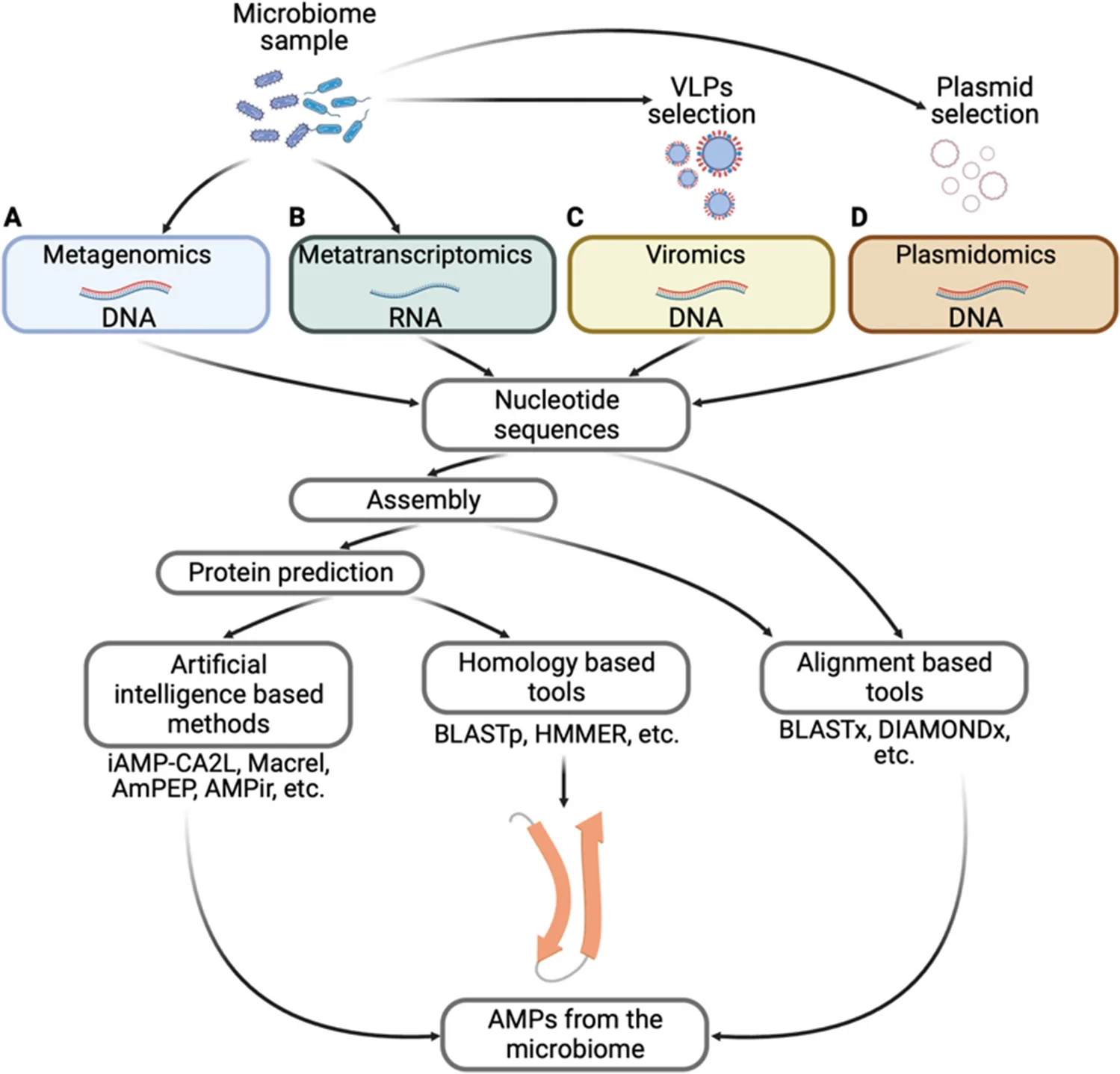
Experimental and bioinformatic strategies to obtain AMPs from microbiome data. The search for AMPs can be done in four kinds of datasets **A **metagenomics, which involves obtaining all potentially functional AMPs, **B **metatranscriptomics focuses on the expressed AMPs, **C** viromics identifies AMPs encoded in viruses, and lastly, **D** plasmidomics is focused on obtaining AMPs codified in plasmids. Image created using BioRender.com

The discovery of new microbiome functionalities has increased with the advances in genomic sciences, including metagenomics, metatranscriptomics, viromics, and plasmidomics [8, 59]. Although the methodologies for nucleic acid extraction, library preparation, and subsequent sequencing are beyond the scope of this review, we briefly describe them. For metagenomics, total DNA is extracted, fragmented, and amplified to create the sequencing libraries (Fig. 6). In contrast, metatranscriptomic libraries require total RNA extraction and enrichment of the molecule of interest (mRNA, lincRNA, microRNA, etc.) and subsequently cDNA synthesis following fragmentation and adapter attachment (Fig. 7) [8]. On the other hand, to study the virome, it is necessary to isolate the viral particles (VLPs). To this end, there are several protocols that use particle-selecting filtration or ultracentrifugation with cesium chloride, followed by DNA or RNA extraction of the enriched VLPs [60]. Finally, to analyze the plasmids of the microbiome, it is necessary the depletion of host bacterial DNA before preparing the sequencing libraries. This can be achieved using exonucleases that degrade linear DNA, leaving the plasmid circular DNA intact [60], then the procedure follows the typical sequencing library protocol (Fig. 7).

After conducting the experimental phase, the next step is to analyze the sequencing data using bioinformatics. When writing this review, only a few articles discussed microbial AMPs obtained from the microbiome (Supplementary File 1). For instance, one study discovered five AMPs produced by the gut microbiota of *Ctenopharyngodon idellus* [61]. Another study found new AMPs derived from the *Hirudo medicinalis* microbiome, identifying a new peptide (pept_1545) that could be used for therapeutic purposes due to its widespread antimicrobial activity and lack of toxicity on eukaryotic cells [62].

A method called Metagenomic AMP Classification and Retrieval (Macrel) found 1263 non-redundant AMPs from 182 human gut metagenomes [63]. More recently, a study used metagenomics data and deep learning to identify AMPs from the human gut microbiome, resulting in the discovery of 2389 AMPs, 181 of which were experimentally proven to have antimicrobial activity [64]. Lastly, using a metagenomics approach, two AMPs (HG2 and HG4) were reported from the rumen microbiome. These peptides showed activity against multidrug-resistant bacteria, making them potentially useful as templates for the treatment of bacterial infections [65].

Metatranscriptomics data led Huang et al. to find microbial AMPs in Taiwanese oolong teas, partially fermented beverages that may impact the microbial communities of the consumer [66]. Another study by Onime et al. found 209 potentially novel AMPs in the rumen of eukaryotic microorganisms using metatranscriptomics. One of these, Lubelisin, was active against methicillin-resistant *Staphylococcus aureus* and maintained low cytotoxicity for humans and sheep [67]. However, as of writing this review, there were no reports of AMPs discovery from viromics or plasmidomics datasets.

## Databases, Web Servers, and Bioinformatics Tools to Discover Microbial AMPs

With the discovery of more AMPs, several research groups have developed publicly available databases (Supplementary File 2). A few examples are YADAMP which contains manually curated AMPs that are effective against bacteria [68]; BACTIBASE, which comprises bacteriocins obtained from bacteria [69]; AntiTbPdb, which has experimentally validated AMPs against tubercular or mycobacterial species [70]; ParaPep, containing Anti-parasitical peptides [71]; AVPdb, with curated Antiviral peptides [72]; InverPep, which includes AMPs produced by invertebrates [73]; and PhytAMP, which only has Plant-derived AMPs [74]. Some databases cater to a particular class of AMPs, like Peptaibol [75] and the Defensins Knowledgebase [76]. Finally, more extensive databases such as ADAM [77] or APD3 [78] offer experimentally validated or manually curated AMPs. Other specialized databases provide detailed information on secondary structures, such as DBAASP [79] or CAMPR3 [80]. Others collect data and remove redundancy while unifying classifications facilitate users to find the AMPs they need, such as dbAMP 2.0 [17], LAMP2 [81], and DRAMP 3.0 [18].

Complementing databases, web servers can help predict if a protein sequence has the potential to be an AMP using various algorithms (Supplementary File 3). However, these web servers have limited capacity for uploading and downloading. Therefore, the massive search for AMPs needs to be optimized data. Furthermore, there are tools available to identify AMPs in a local computer, mostly using a sequence alignment strategy against a protein database, such as BLASTP [82]. Pattern-matching algorithms like Hidden Markov Models (HMM) can also detect remote protein homologs without requiring sequence homology [83]. Although alignment-based methods allow for identifying potential AMPs already reported in databases, they make it challenging to discover new peptides since they depend on already known data. In addition to the sequence alignment methods, other tools consider factors like physic-chemical properties, amino acid composition, and secondary structure to increase the accuracy of the AMPs prediction, such as AMAP [84], AMPir [85], CAMPSign [86], iAMP-2L [87], iAMPred [88], and Macrel [63]. Furthermore, other tools based on artificial intelligence, machine learning, and neural network algorithms can be used to discover AMPs without sequence identity with known AMPs, such as AmPEP [89], amPEPpy 1.0 [90], AMPir [85], APSv2 [91], c_AMP-prediction [64], ClassAMP [92], AMPlify [93], and iAMP-CA2L [94] (Supplementary File 3). These methods allow for finding novel AMPs in genomic data but with the risk of having a higher number of false positives. However, there is no set rule for discovering AMPs from the microbiota, and articles reporting search strategies are scarce. Figure 7 provides a summary of the pipelines for discovering AMPs.

## How to Experimentally Test the AMP Function

**Fig. 8:**
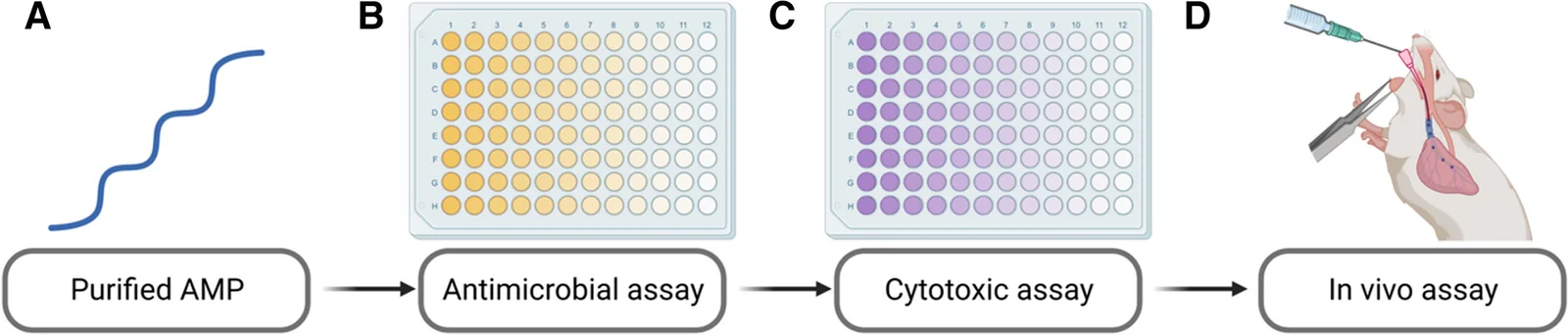
Typical steps for testing the AMP effect for clinical usage. Firstly, peptide purification is necessary, which can be achieved through natural, chemical, or biological synthesis (**A**). Secondly, selecting an appropriate antimicrobial assay is crucial in determining the efficacy of the AMP against the target microorganism (**B**). This involves incubating the AMP and the microorganism together and observing the effects. Additionally, it may be necessary to conduct cytotoxic assays to determine any potential damage to host cells (**C**). Lastly, the AMP can be tested against the target microorganism using an in vivo model (**C**). This can be achieved through various application methods, including topical, intravenous, intraperitoneal, subcutaneous, oral, intranasal, or inhaled. These steps are vital in ensuring the safety and efficacy of AMPs for clinical usage and should be carefully followed to achieve the best possible results. Image created using BioRender.com

To analyze the potential function of an AMP, first, the peptide needs to be obtained by direct extraction from the host, chemical synthesis, or heterologous peptide expression and purification from a bacterial system [95]. After acquiring the peptide, the experimental analysis can be divided into three steps [96]. The first step involves testing the peptide in antimicrobial assays, followed by an in vitro cytotoxic assay, and finally, an in vivo test in case of being used for clinical application [97]. This process is illustrated in Fig. 8.

There are various methods available to test the antimicrobial activity of a peptide. The most common ones are broth microdilution, agar diffusion, agar dilution, and the Kirby-Bauer method. In the Kirby-Bauer method, a filter paper disc is impregnated with the AMP and placed on an agar plate inoculated with the target bacteria [95, 97]. Once the plate has been incubated, the diameter of the inhibition zone is measured. Another helpful test is the minimum inhibitory concentration (MIC) assay, which measures the lowest concentration of a peptide required to inhibit the growth of the target microorganism [97]. In addition to these methods, it is also possible to test the efficacy of an antimicrobial peptide against viruses and fungi and its ability to inhibit cancer cell growth [98, 99].

In the in vitro cytotoxic assays, the process involves incubating the AMP with a suspension of mammalian cells and observing the changes in the cells’ morphology and viability [52]. Live cells are then stained with a specific dye, and the number is compared to those in a control sample that has not been exposed to the peptide [52]. If a lower percentage of cells is stained in the AMP sample, it indicates a higher level of cytotoxicity [52, 100]. The most common method used to determine the cell viability is the MTT assay, which measures the reduction of a yellow MTT (3-(4,5-dimethylthiazol-2-yl)-2,5-diphenyltetrazolium bromide) solution to purple formazan by living cells [52]. Another method is the lactate dehydrogenase assay, which measures the release of LDH from damaged cells [52].

When using AMP as a therapy against a pathogen in the cosmetic or pharmaceutical industries, they must pass an in vivo test [101]. The AMP delivery system to kill the pathogen can be topical, intravenous, intraperitoneal, subcutaneous, oral, intranasal, or inhaled [102]. It is crucial to monitor the animal’s response to the peptide during the assay and adjust the dose as needed [103].

## Future and Perspective of AMPs Derived from Microbiomes

The future of AMPs usage is promising [104], with a wide range of applications that include antibacterial, antifungal, antiviral, and antiparasitic effects [105]. Moreover, researchers are currently exploring their potential use in cancer therapy [106] and as immunomodulators [107]. AMPs are also a safe and well-tolerated natural alternative with few side effects to traditional antibiotics [74], which are becoming less effective due to drug-resistant bacteria [105]. Overall, AMPs represent a hopeful solution to combat infections and diseases with minimal side effects.

The discovery of AMPs traditionally involves screening peptide libraries from the organism of interest to test its antimicrobial activity. This method is largely based on trial-and-error experiments. However, newer approaches use computational methods to predict peptides with antimicrobial activities based on the organism’s proteomic or genomic data. The challenge with this approach is selecting the most suitable organism. Fortunately, the microbiota presents a vast reservoir of undiscovered AMPs that could be clinically and industrially valuable, thanks to its large amount of genomic information, long-term co-evolution with the host, and competition between neighboring bacteria [108]. Therefore, integrating experimental and bioinformatics tools focused on discovering AMPs from metagenomes, metatranscriptomes, viromes, and plasmidomes datasets will be of great value.

The AMPs play a crucial role in limiting the growth of unwanted microbiota, particularly pathogens, and shaping the overall microbiome composition [109]. Understanding AMPs could lead to the development of new therapies that can help regulate the microbiome. These can be used to target diseases caused by microbiota dysbiosis, including skin infections [110], eye diseases [111], gastrointestinal diseases [112], bone and joint infections [113], oral diseases [114], and respiratory diseases [115]. Administering the AMPs using mobile elements as carriers, such as bacteriophages or plasmids, could be a great alternative to combat unwanted microorganisms, like multi-resistant pathogens, without promoting antibiotic resistance [116]. Microbiome-derived AMPs have numerous applications in various industries. They can be used as preservatives to control food-borne pathogens [117]. In agriculture, they can act as growth promoters and control plant diseases [118]. In healthcare, they can treat infections as antiseptics, disinfectants, and drugs [119]. They can control microorganism overgrowth in cosmetics and industrial applications [120, 121].

Besides naturally produced AMPs, the design of new peptides with enhanced antimicrobial activity is an active area of research [122, 123]. This includes improving the antimicrobial activity by modifying the peptide sequence and their cationic, hydrophobic, and amphipathic properties [124], where bioinformatic tools and machine learning or deep algorithms play a crucial role in improving antimicrobial peptides AMPs by aiding in their design, prediction, and analysis [125, 126]. Some of these tools include HydrAMP [127], PepGAN [128], AMPAGAN v2 [129], PepCVAE [130], PandoraGan [131], among others [123].

Challenges are still associated with using AMPs as a solution to antimicrobial resistance. These include issues related to their stability, bioavailability, and production cost [132]. Additionally, further research is needed to determine the optimal dosing and delivery strategies to maximize AMP's effectiveness and minimize the risk of side effects [133].

## Conclusions

The future of AMP research is promising. There are many undiscovered AMPs produced by the microbiota, that are not harmful to the host, presenting a fantastic research opportunity. There is still much to learn about these AMPs, from their discovery and characterization to understanding how they work. Analyzing the omic data from diverse microbiomes and creating new tools and methods for AMP discovery is essential. With all these efforts, the field of AMPs research will make great strides in the coming years.

In light of the growing concern over antimicrobial resistance, AMPs are an encouraging solution against bacteria resistant to multiple antibiotics and diseases linked to microbiota dysbiosis. However, the rapid degradation of peptides in the body often limits their therapeutic potential. Thus, researchers must develop new techniques to enhance AMP delivery and stability. Therefore, it is critical to use AMPs judiciously and deliberately when deploying them on a large scale to address this issue.

Despite these challenges, ongoing research endeavors will be able to confront these obstacles and refine the utilization of AMPs across several industries. Some strategies under exploration include combining AMPs with conventional antibiotics and bacteriophages, developing advanced delivery systems, and designing AMPs with enhanced properties. These efforts aim to unlock the full potential of AMPs for improved functionality.

It is interesting to consider the role of AMPs in host-microorganism interactions. We often think of them as only being produced by the host to fight off harmful bacteria. Nevertheless, it is essential to remember that bacteria can also produce AMPs to defend against the host and compete with other bacteria for resources and survival in the ecological niche. It is a complex dynamic that highlights the intricacies of the microbial world.

## Supplementary Information

Below is the link to the electronic supplementary material.

Supplementary file1 (TIFF 26367 KB) Length distribution of non-microbial AMPs extracted from databases. (A) APD3, with AMPs experimentally validated had 2,856 AMPs not produced by microorganisms, and a median of 28 aa, and 33 aa mean; (B) dbAMP 2.0, a collection of validated and hypothetical AMPs, contained 27,928 non-microbial peptides, with a median of 39 aa, and a mean of 57 aa; (C) DARMP 3.0, a collection of validated and non-validated AMPs, contained 27,149 peptides not produced by microorganisms, with a median of 20 aa and a mean of 24 aa.

Supplementary file2 (XLSX 11 KB)

Supplementary file3 (XLSX 13 KB)

Supplementary file4 (XLSX 12 KB)

## Data Availability

All material relevant to this publication is available in the manuscript and its supplementary information files.
