# Supplementary figures and images for: Perspectives in Searching Antimicrobial Peptides (AMPs) Produced by the Microbiota
Source: Microb Ecol. 2023 Dec 1;87(1):8. doi: 10.1007/s00248-023-02313-8 (PMC10689560; doi:10.1007/s00248-023-02313-8)

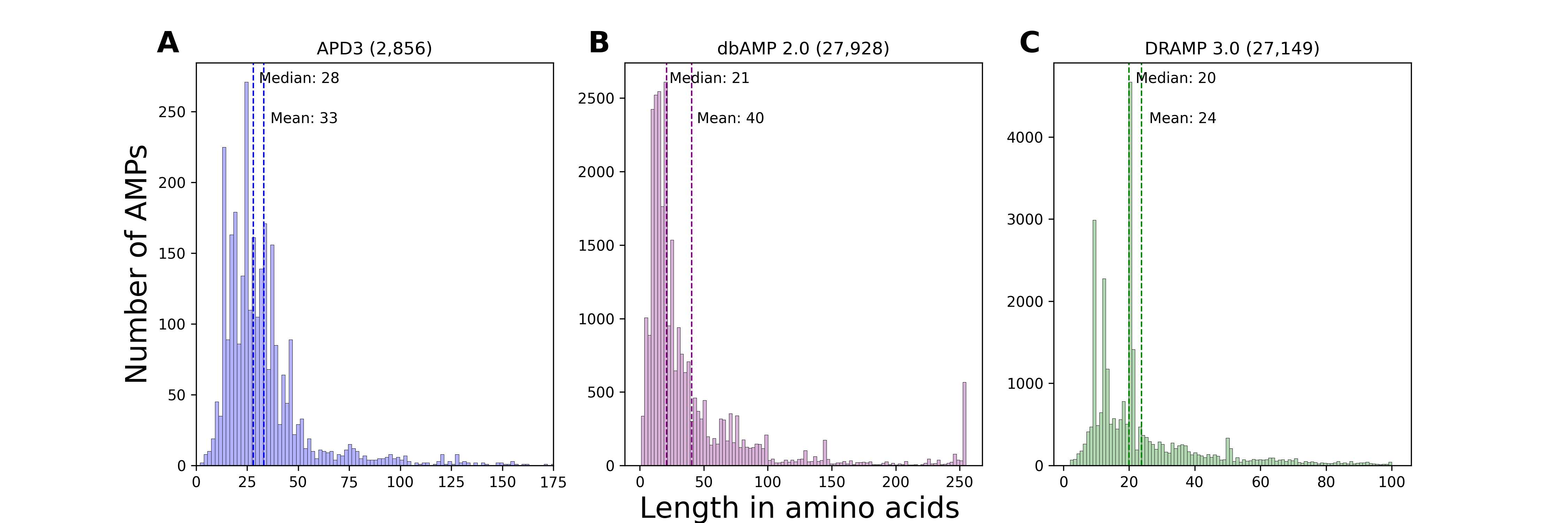

Supplement: Supplementary file 1 — Supplementary file1 (TIFF 26367 KB) Length distribution of non-microbial AMPs extracted from databases. (A) APD3, with AMPs experimentally validated had 2,856 AMPs not produced by microorganisms, and a median of 28 aa, and 33 aa mean; (B) dbAMP 2.0, a collection of validated and hypothetical AMPs, contained 27,928 non-microbial peptides, with a median of 39 aa, and a mean of 57 aa; (C) DARMP 3.0, a collection of validated and non-validated AMPs, contained 27,149 peptides not produced by microorganisms, with a median of 20 aa and a mean of 24 aa. [file 248_2023_2313_MOESM1_ESM.tiff]
